# Trends in the seroprevalence of *Helicobacter pylori* infection and its putative eradication rate over 18 years in Korea: A cross-sectional nationwide multicenter study

**DOI:** 10.1371/journal.pone.0204762

**Published:** 2018-10-17

**Authors:** Seon Hee Lim, Nayoung Kim, Jin Won Kwon, Sung Eun Kim, Gwang Ho Baik, Ju Yup Lee, Kyung Sik Park, Jeong Eun Shin, Hyun Joo Song, Dae-Seong Myung, Suck Chei Choi, Hyun Jin Kim, Jeong Yoon Yim, Joo Sung Kim

**Affiliations:** 1 Departments of Internal Medicine, Healthcare System Gangnam Center Seoul National University Hospital, and Healthcare Research Institute, Seoul, Korea; 2 Department of Internal Medicine, Seoul National University Bundang Hospital, Seongnam, Gyeonggi-do, Korea; 3 Department of Internal Medicine and Liver Research Institute, Seoul National University College of Medicine, Seoul, Korea; 4 College of Pharmacy, Kyungpook National University, Daegu, Korea; 5 Department of Internal Medicine, Kosin University College of Medicine, Busan, Korea; 6 Department of Internal Medicine, Hallym University Medical Center Chuncheon Sacred Heart Hospital, Chuncheon, Gangwon-do, Korea; 7 Department of Internal Medicine, Keimyung University School of Medicine, Daegu, Korea; 8 Department of Internal Medicine, Dankook University Hospital, Cheonan, Chungcheongnam-do, Korea; 9 Department of Internal Medicine, Jeju National University Hospital, Jeju, Jeju-do, Korea; 10 Department of Internal Medicine, Chonnam National University Hwasun Hospital, Hwasun, Jeollanam-do, Korea; 11 Department of Internal Medicine, Wonkwang University College of Medicine, Iksan, Chollabuk-do, Korea; 12 Department of Internal Medicine and Institute of Health Science, Gyeongsang National University School of Medicine, Jinju, Gyeongsangnam-do, Korea; National Cancer Center, JAPAN

## Abstract

The aims of this study were to demonstrate the trends in seropositivity and the eradication therapy rate for *Helicobacter pylori* (*H*. *pylori*) over an 18-year period in an asymptomatic Korean population and to explore the factors associated with *H*. *pylori* seropositivity and its eradication therapy. In total, 23,770 subjects (aged 17–97 years) from a health examination center participated in this cross-sectional study from January 2016 to June 2017. Questionnaires that included questions about the participants’ *H*. *pylori* eradication therapy history were collected, and anti-*H*. *pylori* IgG antibodies were measured. Among the eligible subjects, the seroprevalence of *H*. *pylori* infection was 41.5%. The *H*. *pylori* eradication therapy rate increased continuously from 2005 (13.9%) to 2011 (19.3%) and then increased again until the first half of 2017 (23.5%) (*P* < 0.001). After exclusion of subjects with a history of gastric surgery, gastric cancer, *H*. *pylori* eradication therapy, or gastric symptoms, *H*. *pylori* seropositivity was 43.9% in 16,885 subjects, which was significantly lower than the seropositivities in 1998 (66.9%), 2005 (59.6%), and 2011 (54.4%). The risk factors associated with *H*. *pylori* seropositivity according to multivariable analysis were male sex (odds ratio (OR) 1.34, 95% confidence interval (CI): 1.23–1.46), medium educational level (OR 1.17, 95% CI: 1.05–1.31), medium household income level (OR 1.10, 95% CI: 1.03–1.19), and age of 60–69 years (OR 8.78, 95% CI: 6.41–11.85). The observed downward trend in *H*. *pylori* seroprevalence and increase in *H*. *pylori* eradication over the 18-year period will affect upper gastrointestinal disorders in South Korea.

## Introduction

*Helicobacter pylori* (*HP*) thrives on gastric mucosa in humans and is a major causative factor in peptic ulcer disease and gastritis [1]. It has also been implicated as a factor contributing to gastric adenocarcinoma, gastric mucosa-associated lymphoid tissue lymphoma, and extragastrointestinal diseases, such as cardiovascular diseases, neurological disorders, immunologic impairment, and asthma [1–3].

The prevalence of *HP* infection has declined in recent decades in most countries [4]. Approximately one-third of all adults in Northern Europe and North America are infected with *HP*, and the prevalence is higher than 50% in Africa, Central and South America, Asia, and Southern and Eastern Europe [4]. Consequently, *HP* infection affects more than half the adult population, with some geographical variation in that estimate [5]. The lower prevalence of *HP* infection in developed countries than in developing countries has been attributed to better hygiene and living conditions, which reduce the spread of *HP* [6,7].

In South Korea, studies reported a decrease in the seroprevalence of *HP* infection from 1998 [8] to 2005 [9], and to 2011 [10]. However, the change in the seroprevalence of *HP* infection between 2005 and 2011 was not as large as that between 1998 and 2005 [5]. As South Korea is in a dynamic transition from being a developing country into being a developed country, it may be desirable to evaluate the current seroprevalence of *HP* infection to support the development of health policies to prevent *HP*-related diseases. In addition, understanding provincial trends over time in terms of the prevalence and factors associated with *HP* infection may facilitate effective population-scale healthcare planning worldwide.

Therefore, we hypothesized that the amount of the decrease in *HP* seropositivity could vary provincially over time according to the pace of socioeconomic development. Based on this hypothesis, the aims of this study were to investigate the trends in the seroprevalence and the eradication therapy rate of *HP* infection, and to evaluate the factors associated with *HP* seropositivity over time from 1998 to the first half of 2017 in asymptomatic Korean adults stratified by province.

## Materials and methods

### Study design and population

We conducted a cross-sectional nationwide multicenter study from March 2016 to June 2017 (henceforth abbreviated as 2016–2017) and this study has been written in accordance with the STROBE (Strengthening The Reporting of Observational Studies in Epidemiology) statement guidelines (S1 Table) [11]. A total of 24,471 adult subjects aged 16 years or older visited a healthcare center or an outpatient medical clinic for a routine health examination during the study period and got an esophagogastroduodenoscopy and serologic tests, simultaneously.

**Fig 1:**
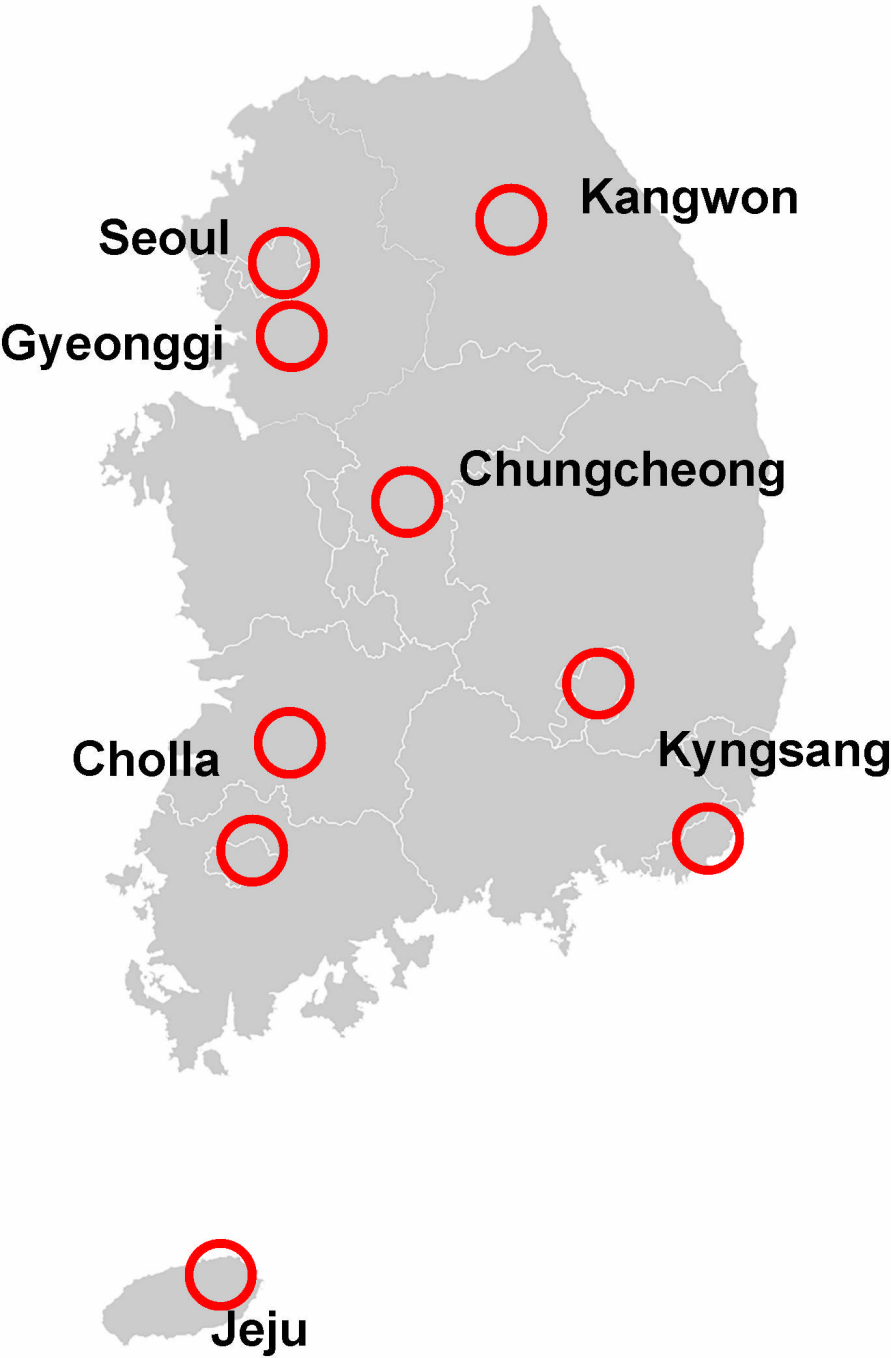
Administrative districts of South Korea and the locations of the 10 healthcare centers that participated in this study (circled).

The 10 healthcare centers that participated in this study were secondary or tertiary academic hospitals located in Seoul and the nine provinces of South Korea, namely, Gyeonggi, Kangwon, North and South Chungcheong, North and South Cholla, North and South Kyungsang, and Jeju (Fig 1). The specific institutions were Seoul National University Hospital Gangnam Center (SNUHGC), which receives subjects from all over the country but mainly from Seoul, the capital of South Korea, where it is located; Seoul National University Bundang Hospital (SNUBH), which is located in Gyeongi-do and serves northwestern South Korea; Hallym University Chuncheon Hospital in Kangwon-do, which serves northeastern South Korea; Kosin University Hospital and Gyeongsang National University Hospital, which serve southeastern South Korea; Chonnam National University Hospital and Wonkwang University Hospital, which serve southwestern South Korea; Dankook University hospital serves central South Korea; and Jeju National University Hospital serves Jeju-do, the largest island off the coast of the Korean peninsula and main island in Jeju Province.

**Fig 2:**
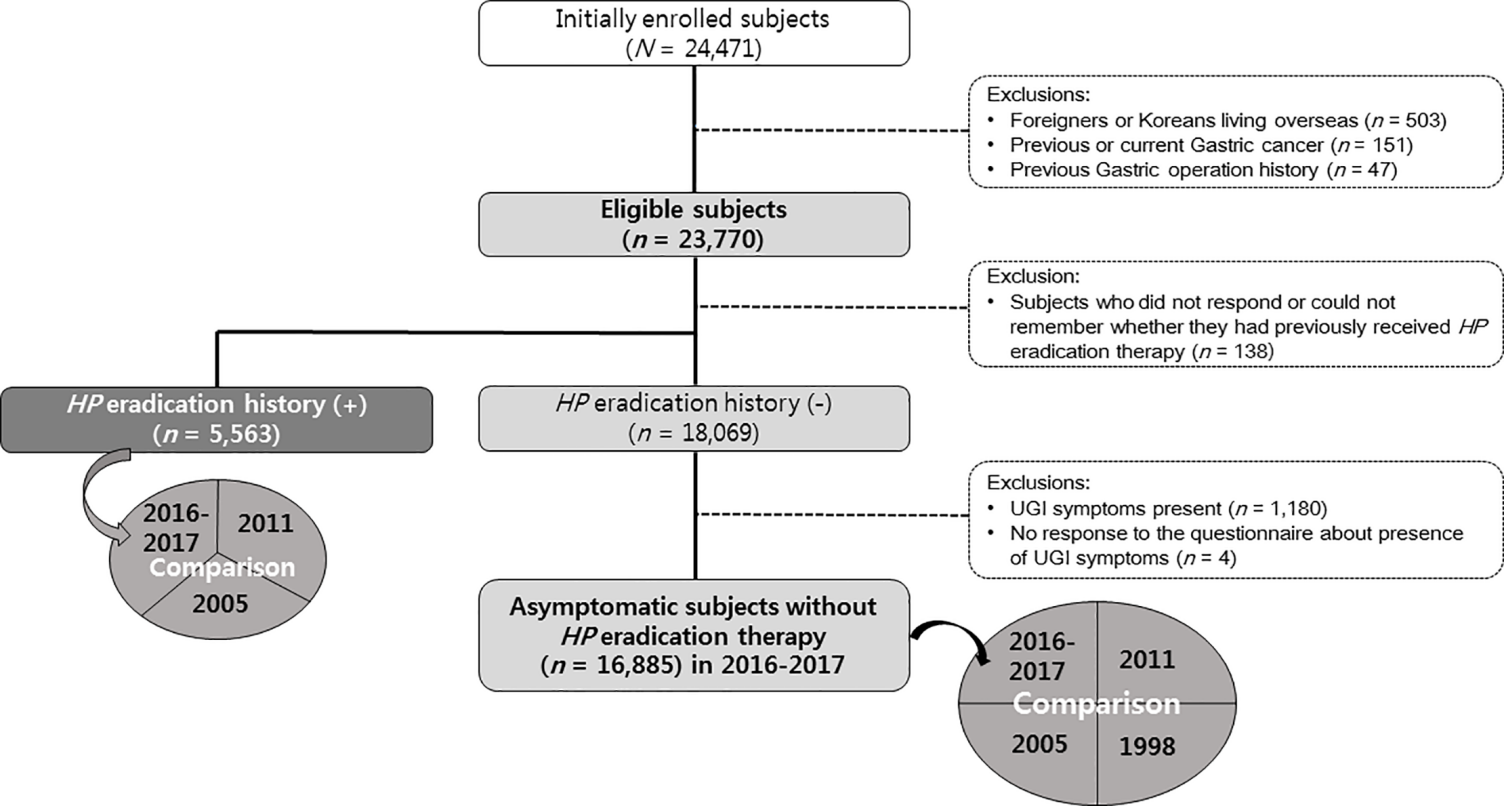
Study flow chart. HP, *Helicobacter pylori*; UGI, upper gastrointestinal.

Among the subjects enrolled prospectively under a predefined protocol, 23,770 were eligible after the exclusion of subjects with a prior history of gastric surgery or gastric cancer (GC) or subjects who were foreigners or Koreans living overseas (Fig 2). Among the 23,770 eligible subjects, 138 subjects who did not respond or could not remember whether they had previously received *HP* eradication therapy, and 5,563 subjects who reported a history of having received *HP* eradication therapy, were excluded in order to avoid uncertainty in the interpretation of the results of the anti *HP-*IgG, and to prevent false positivity or false negativity about *HP* seroprevalence, respectively (Fig 2).

### Data collection

#### Serologic evaluation for *Helicobacter pylori* status

A blood sample was obtained from each participant in the morning after overnight fasting at the same time the interview was conducted, and the serum was separated by centrifugation. The serum was collected and stored according to the same protocol in each hospital. The collected serum from SNUHGC was analyzed at SNUHGC, and all collected serum samples from the nine other provincial hospitals were transferred to and analyzed at the Health Innovation Park of SNUBH.

The diagnosis of *HP* infection was based on the detection of serum *HP* immunoglobulin G antibody (anti-*HP* IgG) using two commercially available immunoassay kits. HPG kits (Immulite 2000 CMIA, Siemens, UK) were used at the SNUHGC, and Genedia *HP* ELISA kits (Green Cross Medical Science Corp., Eumsung, Korea) were used at the remaining centers [10]. The HPG kit used a chemiluminescent enzyme immunoassay and had a sensitivity of 91% and a specificity of 100% [12,13]. Anti-*HP* IgG levels higher than 1.10 IU/mL were regarded as positive in the Immulite 2000 assay, while values in the range from 0 to 1.09 IU/mL were considered negative. The Genedia *HP* ELISA test, which was developed from Korean *HP* strains, had a sensitivity of 97.8% and a specificity of 92% [14] and was previously validated in three nationwide Korean seroepidemiologic studies [8–10].

#### Behavioral factors and previous medical history

All subjects were invited to respond to a questionnaire, which was similar to a questionnaire used in a previous study [10], under the supervision of a well-trained interviewer. The questionnaire requested information regarding demographics (i.e., sex, age, and residence), socioeconomics (i.e., monthly household income and educational level), and medical history (such as *HP* eradication therapy, history of gastric surgery or GC, and smoking habits), and upper gastrointestinal (GI) symptoms (such as indigestion, bloating, or epigastric soreness), that persisted for at least one month within the last 3 years.

The participants were classified according to smoking status as follows: nonsmoker, ex-smoker, or current smoker, with current smoking defined as regular smoking during the previous 12 months. All participants were categorized into the following 7 residential districts according to the geographical location of their residence at the time of examination: Seoul, Gyeonggi, Chungcheong, Kyungsang, Cholla, Kangwon, and Jeju (Fig 1). Each subject was categorized into one of 3 following education levels: low (middle school graduate or less; education duration ≤ 9 years), medium (high school graduate or university dropout; education duration between 10 and 12 years), and high (university graduate or postgraduate; education duration ≥ 13 years). The participant’s monthly household income was classified into one of the 3 following groups: low (<3,000 US dollars per month), medium (3,000 to 10,000 US dollars per month), and high (>10,000 US dollars per month).

### Statistical analysis

Dependent variable was *HP* seropositivity, and independent variables were all the other variables such as sex, age group, resident area, or socioeconomic factors in this study. Student’s t-test or the Mann-Whitney test for continuous variables and the chi-squared test for categorical variables were used to analyze differences in demographic and clinical variables according to *HP* seropositivity. To explore the factors associated with *HP* seropositivity and the factors associated with a history of *HP* eradication therapy, multivariable logistic regression was used. The odds ratios (OR) and 95% confidence intervals (CI) were calculated. A two-tailed *P*-value < 0.05 was considered statistically significant. The trends in the *HP* seroprevalence in the periods between 1998, 2005, 2011, and 2016–2017 were compared using the published data of 1998 [8], 2005 [9], and 2011 [10], with the data restricted to asymptomatic subjects without a history of *HP* eradication therapy or gastric operation. The statistical comparison of the trends in the *HP* seroprevalence between 1998, 2005, 2011, and 2016–2017 was conducted using the Cochrane-Armitage trend test, which is a modified Pearson chi-squared test that analyzes the association between a binary outcome and a variable with multiple categories. All statistical analyses were performed with SPSS (SPSS 22.0J, IBM, New York, USA) and the DescTools packages in R version 3.4.3.

### Ethics statement

Written informed consent was obtained from each participant or the participant’s parent.

This study was approved by the Institutional Review Board for Seoul National University Hospital (IRB No. H-1602-057-740).

## Results

### Seroprevalence of *HP* infection in all eligible subjects

**Fig 3:**
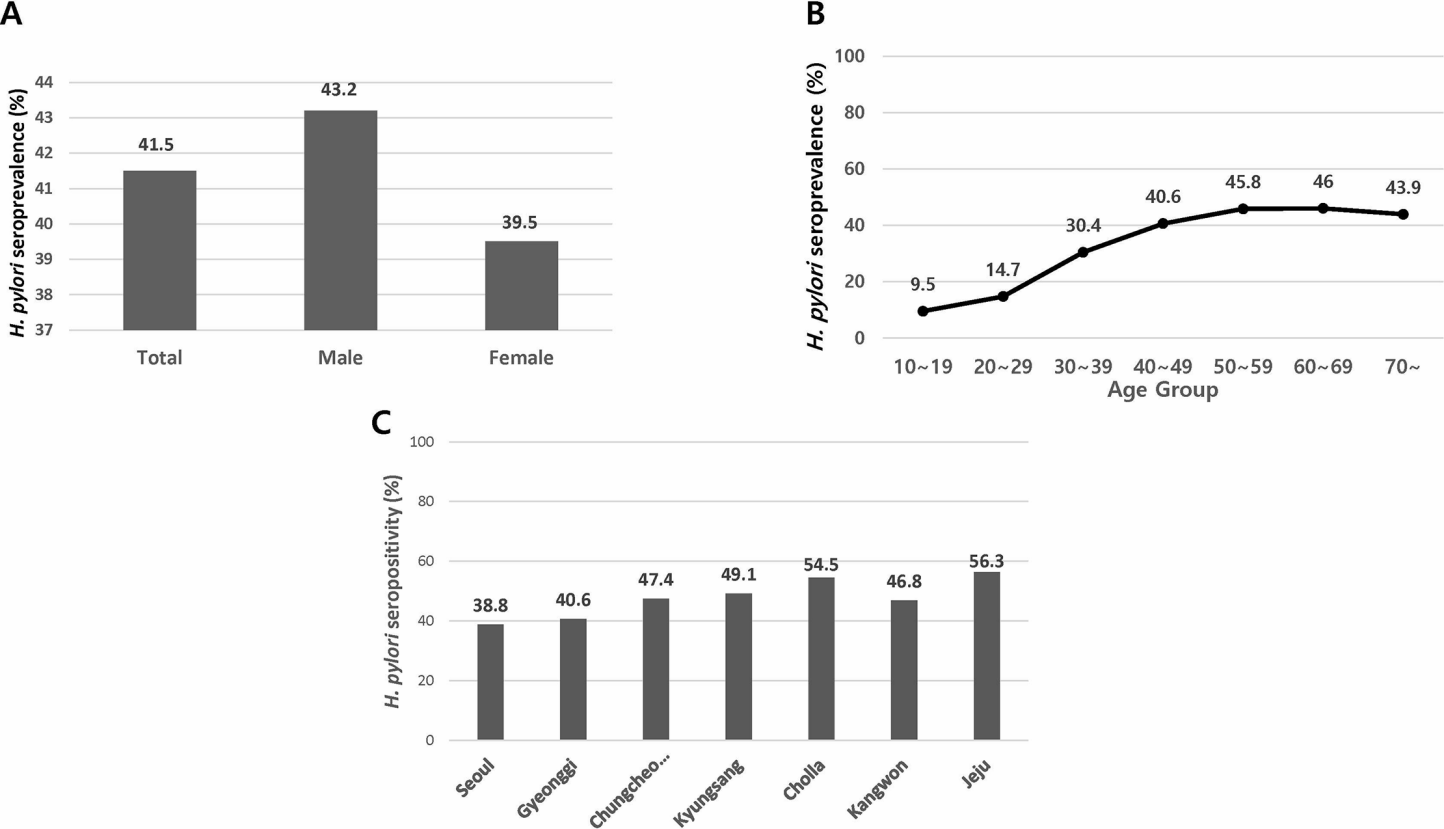
Seroprevalence of *Helicobacter pylori* among eligible subjects in 2016–2017 stratified by gender (A), age group (B), and geographical area (C).

In total, there were 23,770 eligible subjects; the mean age of the eligible subjects was 51.7±11.6 years, and 41.5% (9,871/23,770) of the subjects were seropositive for anti-*HP* IgG. Table 1 shows the demographic and clinical characteristics of the participants as well as the seroprevalence of each group. The seroprevalence of *HP* infection was significantly higher in males than in females (43.2% vs. 39.5%, *P* < 0.001) (Fig 3A). Seropositivity tended to increase with age, although it decreased slightly in the older age group (*P* < 0.001); seropositivity increased from 9.5% in subjects aged 16–19 years to 46.0% in subjects aged 60–69 years and then decreased to 43.9% in the group older than 70 years (Fig 3B). When the data were stratified according to geographical residence, Seoul had the lowest seroprevalence of *HP* infection (38.8%), followed by Gyeonggi (40.6%). Most geographical areas, with the exception of Cholla and Jeju, had anti-*HP* IgG prevalence values less than 50% (Fig 3C). There was no difference in *HP* seropositivity between participants with and without upper GI symptoms (41.5% vs 41.6%, *P* = 0.954) (Table 1).

**Table 1:** Baseline characteristics of the 23,770 eligible subjects.

		Total subjects (No)	*H*. *pylori* seropositive subjects (No, [%])
**All**		23,770	9,871 [41.5]
**Gender**	Male	12,972	5,610 [43.2]
	Female	10,798	4,261 [39.5]
**Mean age±SD (years)^†^**		51.7±11.6	53.3±10.5
**Age group (years)**	16–19	21	2 [9.5]
	20–29	763	112 [14.7]
	30–39	2,726	830 [30.4]
	40–49	6,326	2,567 [40.6]
	50–59	8,115	3716 [45.8]
	60–69	4,287	1972 [46.0]
	≥70	1,532	672 [43.9]
**Geographical areas**	Seoul	14,270	5535 [38.8]
	Gyeonggi	4,258	1,729 [40.6]
	Chungcheong	851	403 [47.4]
	Kyungsang	2,760	1,354 [49.1]
	Cholla	727	396 [54.5]
	Kangwon	579	271 [46.8]
	Jeju	325	183 [56.3]
**House income^‡^**	Low	1,605	741 [46.2]
	Medium	9,157	3,901 [42.6]
	High	11,589	4,650 [40.1]
	missing	1,416	579
**Educational level^§^**	Low	1,027	516 [50.2]
	Medium	3,331	1,500 [45.0]
	High	19,186	7,746 [40.4]
	missing	226	109

Subjects with missing values were excluded; No, number; *H*. *pylori*, *Helicobacter pylori*; M, male; F, female; UGI, upper gastrointestinal

^†^mean ± standard deviation

^‡^Household income was classified as low (less than US $ 3,000 per month), medium (US $ 3,000 to 10,000 per month), or high (more than US $ 10,000 per month)

^§^Educational level was classified as low (middle school graduates or less), middle (high school graduates or university dropouts), or high (university graduates or graduates of a postgraduate course).

### History of *HP* eradication therapy

**Fig 4:**
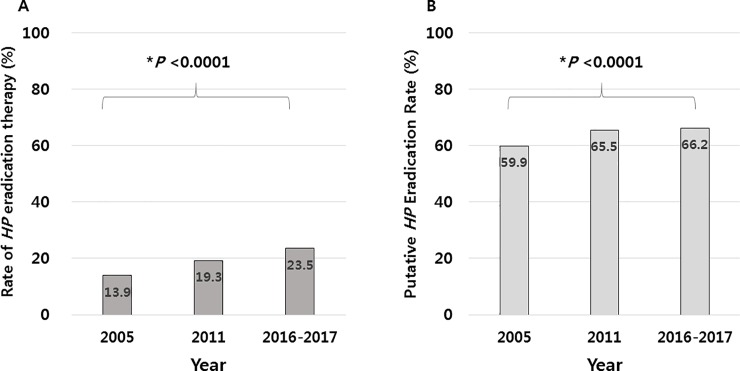
Eradication history of *Helicobacter pylori* (*HP*) in 2005, 2011, and 2016–2017. **(A) Comparison of the *HP* eradication therapy rate among eligible subjects. (B) Putative eradication rate according to seroconversion among the subjects who received *HP* eradication therapy in 2005, 2011, and 2016–2017.** (*trend *P*-value).

**Fig 5:**
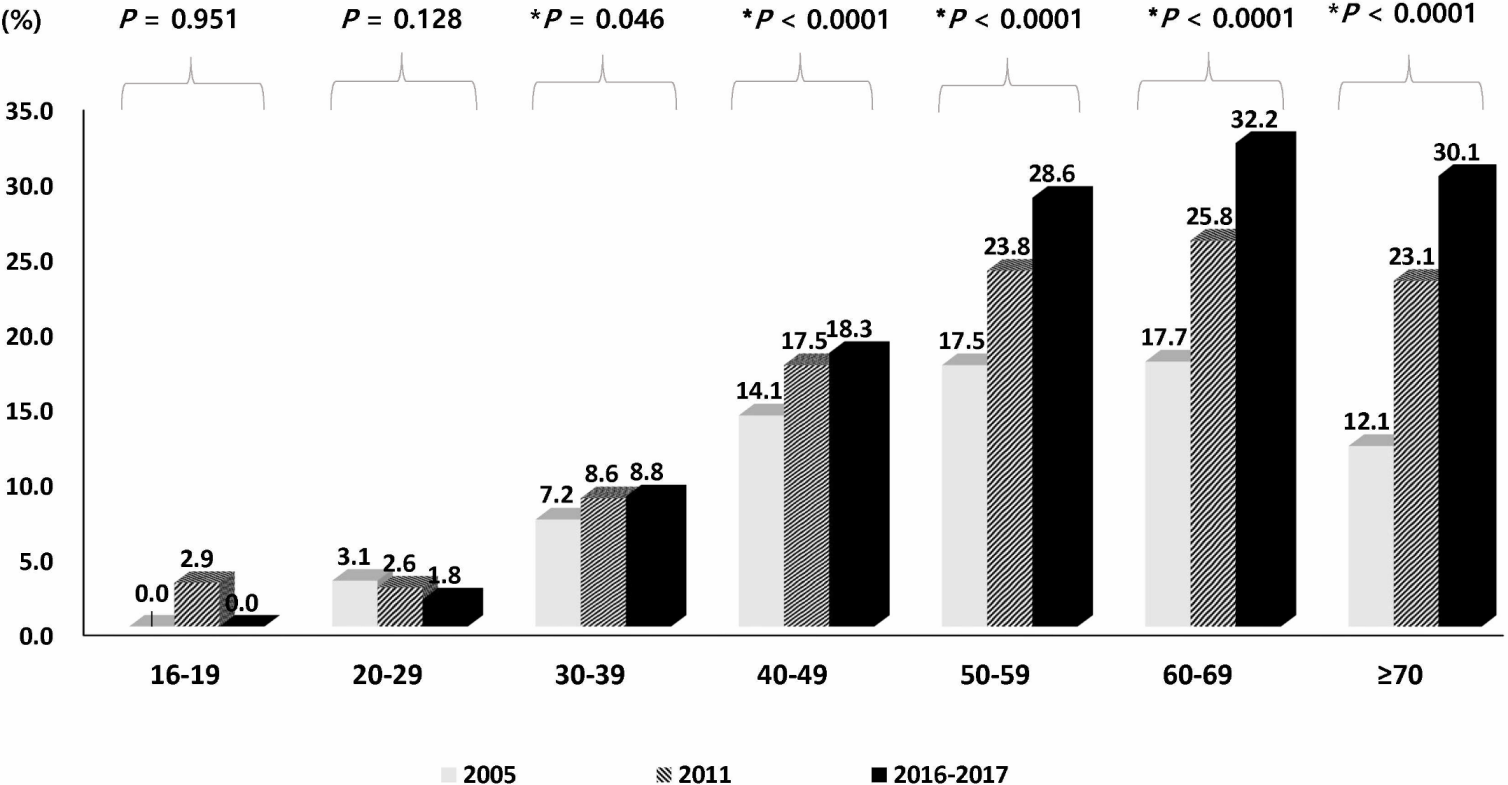
Trends in the rate of *Helicobacter pylori* eradication therapy stratified by age group in 2005, 2011, and 2016–017. (*trend *P*-value).

**Fig 6:**
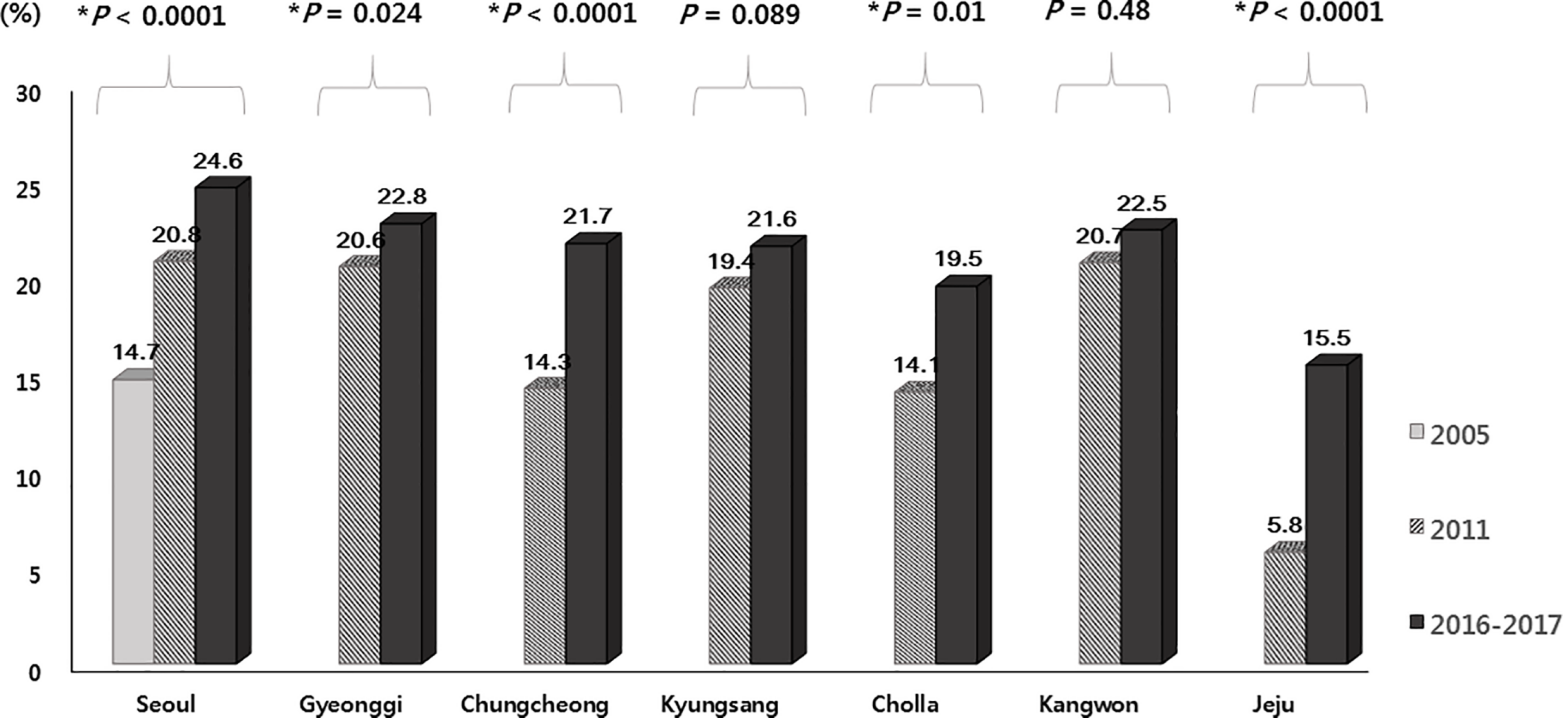
Trends in the rate of *Helicobacter pylori* eradication therapy stratified by geographical area in 2005, 2011, and 2016–2017. (*trend *P*-value).

Among the 23,770 eligible subjects, 138 subjects did not respond or could not remember whether they had previously received *HP* eradication therapy, and were excluded to avoid uncertainty in the interpretation of the results of the anti-*HP* IgG test. Of remaining subjects, 5,563 (23.5%, 5,563/23,632) reported a history of having received *HP* eradication therapy, regardless of the outcome of therapy (henceforth, “the proportion of subjects who received HP eradication therapy” is termed “the *HP* eradication therapy rate” to avoid confusion with “the *HP* eradication rate”) (Fig 2). A comparative analysis of *HP* eradication therapy rate among the eligible subjects in 2005, 2011, and 2016–2017 was performed, and the data from 2005 [9] and 2011 [10] were explored (Table 2 & Figs 4–6). Overall, 23.5% of the eligible subjects received *HP* eradication therapy in 2016–2017, which was significantly increased from 19.3% in 2011, which in turn was increased from 13.9% in 2005 (*P* < 0.05 in each comparison), demonstrating a significant upward trend in the of *HP* eradication therapy rate over the 11 years from 2005 to 2016–2017 (trend *P* < 0.0001) (Fig 4A).

**Table 2:** Prevalence of *Helicobacter pylori* eradication therapy.

	2005 No [%]	2011 No [%]	2016–2017 No [%]	Trend *P*-value
**Eligible subjects**	15,916	19,272	23,632	
**Male**	8,616	10,557	12,897	
**Residents in Seoul**	10,312	10,755	14,266	
**Subjects who had taken *HP* eradication therapy** **[the rate of *HP* eradication therapy, %]**	2,219 [13.9]	3,712 [19.3]	5,563 [23.5]	**< 0.0001**
**Gender**				
**male**	1,331 [15.4]	2,246 [21.3]	3,443 [26.7]	**< 0.0001**
**female**	888 [12.2]	1,466 [16.8]	2,120 [19.7]	**< 0.0001**
**Geographic area**				
**Seoul**	1,516 [14.7]	2,240 [20.8]	3,516 [24.6]	**< 0.0001**
**Gyeonggi**	5,603* [12.5]	622 [20.6]	969 [22.8]	**0.024**^‡^
**Chungcheong**		123 [14.3]	184 [21.7]	**< .0001**^‡^
**Kyungsang**		317 [19.4]	588 [21.6]	0.089^‡^
**Cholla**		254 [14.1]	137 [19.5]	**0.001**^‡^
**Kangwon**		122 [20.7]	124 [22.5]	0.482^‡^
**Jeju**		34 [5.8]	45 [15.5]	**< 0.0001**^‡^
**Age group**				
**16–19**	0 [0]	1 [2.9]	0 [0]	0.951
**20–29**	25 [3.1]	21 [2.6]	14 [1.9]	0.128
**30–39**	180 [7.2]	246 [8.6]	237 [8.8]	**0.046**
**40–49**	678 [14.1]	889 [17.5]	1,153 [18.3]	**< 0.0001**
**50–59**	860 [17.5]	1,467 [23.8]	2,311 [28.6]	**< 0.0001**
**60–69**	415 [17.7]	865 [25.8]	1,385 [32.5]	**< 0.0001**
**≥70**	61 [12.1]	223 [23.1]	463 [30.4]	**< 0.0001**
**Subjects who were anti-*HP* IgG negative in subjects who had *HP* eradication therapy history [putative *HP* eradication rate, %]**	1,330 [59.9]	2,430 [65.5]	3,680 [66.2]	**< 0.0001**

HP, Helicobacter pylori

*, the total number for 6 provincial geographic areas

^‡^, each trend p value for comparison of *HP* eradication therapy rate between two periods, 2011 & 2016–2017.

Bold font indicates statistical significance.

Serological testing of the 5,563 subjects demonstrated that 66.2% (3,680/5,563) were negative for anti-*HP* IgG, and 33.8% were positive. We also found that among the subjects who had previously undergone *HP* eradication therapy in each study, the percent of the subjects who were seronegative for *HP* increased from 59.9% in 2005 to 66.2% in 2016–2017 (trend *P* < 0.0001) (Fig 4B). The rate of *HP* eradication therapy increased with increasing age, except in two age groups (16–19 and 20–29 years) (Fig 5). When the data were stratified according to geographical area, the rate of *HP* eradication therapy showed significant increasing trends in most areas over time, although the changes in two areas, Kyungsang and Kangwon, failed to reach statistical significance (Fig 6).

### Factors affecting *HP* eradication

Logistic regression analysis of the factors influencing whether a subject received *HP* eradication therapy showed that those subjects aged 60 years and older were more likely to have received therapy than those aged 20–29 (OR 22.29, 95% CI: 13.06–38.04, *P* < 0.0001), those residing in Seoul were more likely to have received therapy than those residing elsewhere in South Korea (OR 1.81, 95% CI: 1.30–2.52, *P* < 0.0001), those with GI symptoms were more likely to have received therapy than those without GI symptoms (OR 1.6, 95% CI: 1.41–1.81, *P* < 0.0001), males were more likely to have received therapy than females (OR 1.4, 95% CI: 1.31–1.5, *P* < 0.0001), those with higher levels of household income were more likely to have received therapy than those with lower levels of household income (OR 1.25, 95% CI: 1.06–1.46, *P* = 0.007), and those who were smokers were more likely to have received therapy than those who were nonsmokers (OR 1.15, 95% CI:1.02–1.29, *P* = 0.022) (Table 3).

**Table 3:** Multiple regression analyses of the factors affecting *Helicobacter pylori* eradication therapy.

Variable category		Total (No)	*HP* therapy (No, [%])	Multivariable OR	analysis 95% CI	*P*-value
**All**		23,632^†^	5,563 [23.5]			
**Gender**	Male	12,897	3,443 [26.7]	1.40	1.31–1.50	**< 0.0001**
	Female	10,735	2,120 [19.7]	ref		
**Age group**	16–19	19	0 [0]	0	0	0.999
	20–29	747	14 [1.9]	ref		
	30–39	2,708	237 [8.8]	4.29	2.48–7.43	**< 0.0001**
	40–49	6,296	1,153[18.3]	9.90	5.80–16.89	**< 0.0001**
	50–59	8,081	2,311 [28.6]	17.92	10.52–30.55	**< 0.0001**
	60–69	4,259	1,385 [32.5]	22.29	13.06–38.04	**< 0.0001**
	≥ 70	1,522	463 [30.4]	20.41	11.87–35.12	**< 0.0001**
**Geographical areas**	Seoul	14,266	3,516 [24.6]	1.81	1.30–2.52	**< 0.0001**
	Gyeonggi	4,255	969 [22.8]	1.61	1.15–2.26	**0.006**
	Chungcheong	846	184 [21.7]	1.40	0.97–2.03	0.074
	Kyungsang	2,721	588 [21.6]	1.59	1.13–2.24	**0.009**
	Cholla	701	137 [19.5]	1.17	0.80–1.71	0.431
	Kangwon	552	124 [22.5]	1.51	1.01–2.27	**0.045**
	Jeju	291	45 [15.5]	ref		
House income^‡^	Low	1,555	308 [19.8]	ref		
	medium	9,081	1,988 [21.9]	1.15	0.98–1.35	0.078
	High	11,584	2,988 [25.8]	1.25	1.06–1.46	**0.007**
**UGI symptoms**	Absence	21,980	5,095 [23.2]	ref		
	Presence	1,648	468 [28.4]	1.60	1.41–1.81	**< 0.0001**
**Smoking**	Nonsmoker	17,333	3,856 [22.2]	ref		
	Ex-smoker	3,967	1,172 [29.5]	1.13	1.04–1.24	**0.006**
	Current smoker	2,294	525 [22.9]	1.15	1.02–1.29	**0.022**

Subjects with missing values in the previous *H*. *pylori* eradication therapy were excluded. Odds ratio (OR) was mutually adjusted for all factors. *HP*, *Helicobacter pylori*; No, number; CI, confidence interval; UGI, upper gastrointestinal

^†^Among the 23,770 eligible subjects, 138 who failed to respond to the questionnaire surveying previous H. pylori eradication therapy or who were unsure whether they had received *H*. *pylori* eradication therapy were excluded

^‡^Household income was classified as low (less than US $ 3,000 per month), medium (US $ 3,000 to 10,000 per month), or high (more than US $ 10,000 per month).

Bold font indicates statistical significance.

### Seroprevalence and factors associated with *HP* seropositivity in asymptomatic subjects without a history of *HP* eradication therapy

Among the 23,770 eligible subjects, 16,885 subjects were asymptomatic and had no history of having received *HP* eradication therapy (Fig 2). The seroprevalence of *HP* infection among these study subjects was 43.9% (7,416/16,885). Males had a higher seroprevalence of *HP* infection than females (47.1% vs 40.4%, *P* < 0.0001) (Table 4). When the data were stratified according to geographical area, it was revealed that the seroprevalence of *HP* infection was 41.4% in Seoul, 43.0% in Gyeonggi, 50.3% in Chungcheong, 50.6% in Kyungsang, 57.4% in Cholla, 47.8% in Kangwon, and 61.5% in Jeju (Table 4).

**Table 4:** Factors associated with *Helicobacter pylori* seropositivity in 16,885 asymptomatic subjects without a history of receiving *H*. *pylori* eradication and gastric operation.

Variable category		Total	Seropositivity	Multivariable Analysis		
		No	No [%]	OR	95% CI	*P*-value
**All**		16,885	7416 [43.9]			
**Gender**	Male	8,950	5262 [47.1]	1.34	1.23–1.46	**< 0.0001**
	Female	7,935	3857 [40.4]	ref		
**Age Group (years)**	16–19	17	1 [5.9]	0.000	0.000	0.999
	20–29	630	86 [13.7]	ref		
	30–39	2,263	645 [28.5]	2.73	2.00–3.72	**< 0.0001**
	40–49	4,870	2021 [41.5]	5.33	3.95–7.21	**< 0.0001**
	50–59	5,478	2725 [49.7]	7.32	5.42–9.88	**< 0.0001**
	60–69	2,656	1219 [54.1]	8.78	6.41–11.85	**< 0.0001**
	≥70	971	501 [51.6]	7.30	5.24–10.15	**< 0.0001**
**Geographical areas**	Seoul	10,189	4222 [41.4]	ref		
	Gyeonggi	3,142	1350 [43.0]	0.98	0.90–1.07	0.66
	Chungcheong	595	299 [50.3]	1.31	1.07–1.61	**0.009**
	Kyungsang	1,906	965 [50.6]	1.45	1.27–1.64	**< 0.0001**
	Cholla	502	288 [57.4]	1.24	0.95–1.60	0.11
	Kangwon	343	164 [47.8]	1.57	1.04–2.35	**0.03**
	Jeju	208	128 [61.5]	1.62	0.95–2.76	0.076
**Household income level** ^†^	Low	1,066	491 [46.1]	1.19	0.99–1.42	0.07
	Medium	6,612	2965 [44.8]	1.10	1.03–1.19	**0.006**
	High	8,200	3542 [43.2]	ref		
**Educational level**^‡^	Low	640	339 [53.0]	1.00	0.99–1.26	0.995
	Medium	2,269	1105 [48.7]	1.17	1.05–1.31	**0.006**
	High	13,829	5900 [42.7]	ref		
**Smoking**	Nonsmoker	12,538	5425 [43.3]	ref		
	Ex-smoker	1,656	705 [42.6]	0.96	0.84–1.08	0.47
	Current smoker	2,673	1276 [47.7]	0.97	0.86–1.05	0.34

Subjects with missing values were excluded. Odds ratio (OR) was mutually adjusted for all factors.

*HP*, *Helicobacter pylori*; No, number; OR, odds ratio; CI, confidence interval

^†^Household income was classified as low (less than US $ 3,000 per month), medium (US $ 3,000 to 10,000 per month), or high (more than US $ 10,000 per month)

^‡^Educational level was classified as low (middle school graduates or less), medium (high school graduates or university dropouts), or high (university graduates or graduates of a postgraduate course). Bold font indicates statistical significance.

Multivariable analysis of the factors related to *HP* seropositivity in asymptomatic subjects without a history of *HP* eradication therapy showed that there were significant associations between seropositivity and sex, age, geographical area, economic status, and educational level (Table 4). Males had a higher likelihood of *HP* seropositivity than females (OR = 1.34, 95% CI: 1.23–1.46, *P* < 0.0001). Analysis of the data by age group revealed that the likelihood of *HP* seropositivity increased in a nearly linear fashion from subjects aged 30–39 years to those aged 60–69 years but that the likelihood of *HP* seropositivity was slightly lower in those older than 70 years than in those aged 20–29 years. Subjects aged 20–29 years showed the lowest *HP* seropositivity (13.7%) and this increased up to 54.1% in those aged 60–69 years (OR 8.78, 95% CI: 6.41–11.85, *P* < 0.001) (Table 4). Subjects living in Seoul had a lower likelihood of *HP* seropositivity than subjects living in the other provinces, except for Gyeonggi. However, the statistical significance of the positive association with *HP* seropositivity in Cholla and Jeju disappeared when adjusted for other confounders. Subjects with lower (low and medium) household income levels and lower (low and medium) educational levels had a higher likelihood of *HP* seropositivity compared with those with high levels of income and education. However, statistical significance was only observed in subjects with medium income and medium educational levels (Table 4).

### Comparison of *HP* seroprevalence in asymptomatic subjects without a history of *HP* eradication therapy in 2016–2017, 2011, 2005, and 1998

**Fig 7:**
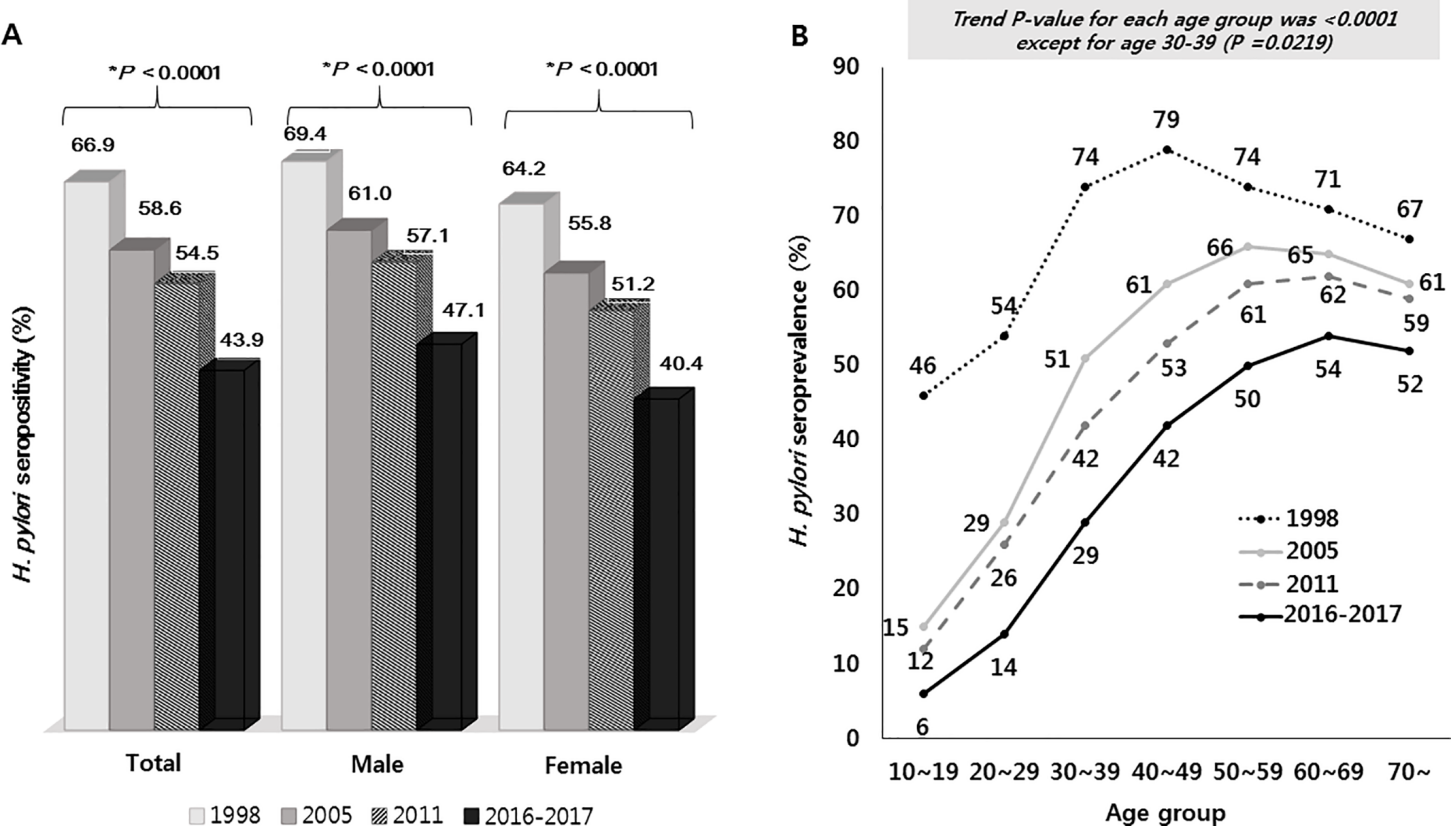
**Trends in the seroprevalence of *Helicobacter pylori (HP)* infection in asymptomatic subjects without a history of receiving *HP* eradication therapy stratified by sex (A) and age (B) in 1998, 2005, 2011, and 2016–2017.** (*trend *P* < 0.05).

The *HP* seroprevalence was compared based on the data from 1998 study [8] by the Korean *HP* Study Group, and from 2005 [9], 2011 [10], and 2016–2017 studies by our group. The overall *HP* seropositivity infection was 43.9% in 2016–2017, which was markedly decreased from 54.4% in 2011, 59.6% in 2005, and 66.9% in 1998 (trend *P* < 0.001) (Fig 7A). There were statistically significant reductions in seropositivity from 2011 to 2016–2017, from 2005 to 2011, and from 1998 to 2005. Each of the four studies demonstrated that males had a higher seropositivity of *HP* infection than females and that seropositivity decreased continuously over time in each sex (Fig 7A).

**Fig 8:**
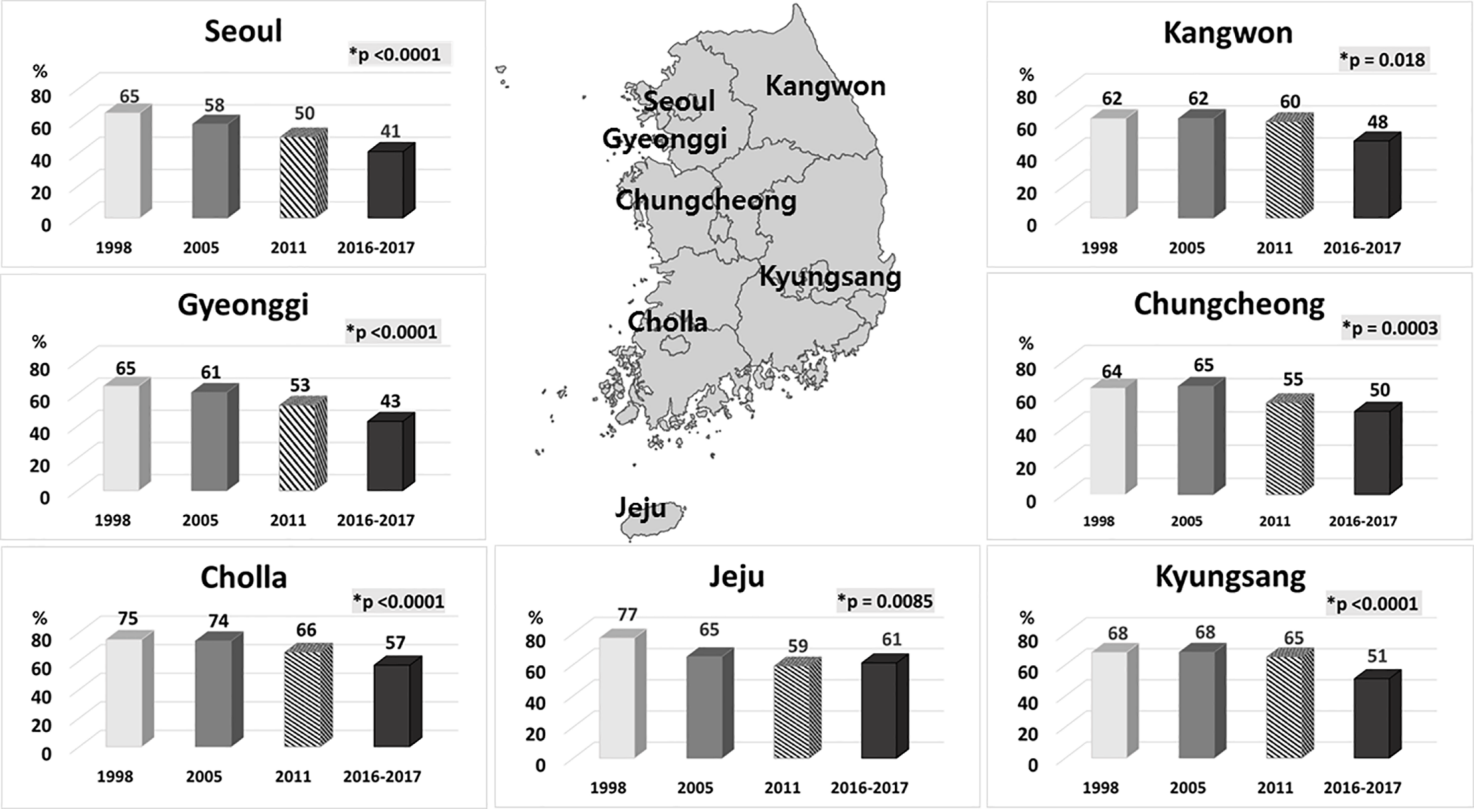
Trends in the seroprevalence of *Helicobacter pylori (HP)* infection in asymptomatic subjects without a history of receiving *HP* eradication therapy stratified by geographical area in 1998, 2005, 2011, and 2016–2017. (*trend *P* < 0.05).

The *HP* seroprevalence decreased significantly in all age groups from 1998 to 2016–2017 (trend *P* < 0.05) (Fig 7B). Out of all the age groups assessed, the largest decrease in seropositivity was observed in the group aged 30–39 years (from 74–29%) and the smallest was detected in the group aged 70 years and older (from 67–52%). In contrast with what was observed between 2005 and 2011, a nearly consistent decrease in seroprevalence was detected across all age groups between 2011 and 2016–2017. The prevalence of seropositivity showed a significant downward trend in all geographical areas over time (each trend *P* < 0.05), although the seropositivity in Jeju increased slightly in 2016–2017 (61.5%) compared with that in 2011 (58.9%) (Fig 8).

## Discussion

In this large nationwide cohort study in South Korea, the *HP* seroprevalence was 43.9% in adults aged ≥ 16 years, and 23.5% of participants had received *HP* eradication therapy. In addition, four Korean *HP* prevalence studies using serum anti-*HP* IgG in 1998 [8], 2005 [9], 2011 [10], and 2016–2017 showed a clear decrease in the prevalence from 66.9% to 43.9% over time, regardless of age, sex, or geographical area. This decline may be attributed to the changes associated with higher socioeconomic status, better sanitary conditions, and greater distribution of nuclear family [4,5,15]. In addition, an individual’s dietary habits, income spent on healthcare, and environmental factors other than the factors identified in this study, may influence the *HP* seroprevalence; the current study could not explore these potentially contributing factors.

The observed decrease in the seroprevalence of *HP* infection over time is in agreement with the decline in the prevalence of *HP* infection in developed countries reported in a recent systemic review [16] of the global prevalence of *HP* infection. Hooi et al. reported that the prevalence of *HP* has been declining in the highly industrialized countries of the Western world, whereas the prevalence plateaued at a high level in developing and newly industrialized countries, at the turn of the 21st century [16]. Although the decline in seroprevalence over time may have resulted from the birth-cohort phenomenon among different generations [4,17], spontaneous or unintended eradication of *HP* during that period may have partly contributed to the decrease in prevalence observed in the current study.

The seroprevalence of *HP* infection in this study increased gradually with increasing age, as in previous studies [8,9]. However, a very low seropositivity (6%) was observed in subjects aged 16–19 years, which was similar to the pattern observed in developed countries [4,17]. The increasing prevalence with increasing age observed in this study is largely explained by the birth-cohort effect, with decreasing colonization rates in subsequent generations [4,15,17]. The decrease in seropositivity observed in subjects older than 70 years after the peak in the previous decade may be explained by the loss of *HP* following the development of atrophic gastritis, following coincidental treatment with antibiotics, or following the development of immunity without extensive host injury [17].

In terms of the geographical area, an overall declining trend in seropositivity was observed in all areas, including Kyungsang and Kangwon, which had showed no significant decline between 2005 and 2011. In contrast to the overall trend, the seroprevalence in Jeju increased from 59% in 2011 to 61% in 2016–2017; this increase might have reflected an influx of population into the area, stochastic variation, or an increasing incidence of *HP* in adults on this island. Jeju is the province in Korea preferred by many elderly, especially after retirement. Therefore, there may be a high probability of an influx of seniors into Jeju Province, recently. In practice, in Jeju Province, the seropositivity among subjects aged 50–59 and 60–69 was 66.7% and 82.5% in 2016–2017, respectively, compared with 59.0% and 52.0% in 2011, respectively (S1 Fig).There have been many studies about the *HP* eradication (success) rate; however, to the best of our knowledge, there have been no published reports, other than studies performed by our group [9,10] about the rate of *HP* eradication therapy, i.e. the proportion of subjects who received *HP* eradication therapy regardless of the outcomes of eradication, which is considered an important index to clarify the current clinical situation. The rate of *HP* eradication therapy in the current study, regardless of the outcome, was 23.5%, which was significantly higher than the 13.9% reported in 2005, and this number has increased over time from 2005 to 2016–2017 (trend *P* < 0.05). The factors underlying this increase may not be due to an increased incidence of *HP*-related diseases, including peptic ulcer diseases (PUD), which are the main indication for *HP* eradication therapy, but rather due to easier access to medical information and medical services and/or change over time in the guidelines defining the target population for *HP* eradication therapy in South Korea. In practice, one study from Korea reported that the prevalence of *HP* infection in patients with PUD was 68.1% in 1995, 59.7% in 2000, and 57.2% in 2005, while the prevalence of PUD was 18.0% in 1995, 19.1% in 2000, and 20.2% in 2005 (*P* < 0.001) [18]. The uses of non-steroidal anti-inflammatory drugs has replaced *HP* infection as the main cause of PUD, partly due to an increase in the aging population [19,20]. Moreover, there have been three guideline releases for the diagnosis and treatment of *HP* infection by Korean College of *Helicobacter* and Upper Gastrointestinal Research (formerly the Korean *H*. *pylori* study group or the Korean Society of *Helicobacter* and Upper Gastrointestinal Research) in South Korea. After the first release of the consensus regarding the target population for the treatment of *HP* infection in 1998 [21], new treatment guidelines were published in 2009 [22] based on updated research and literature, and then a further updated version was issued in 2013 [23] that had been revised on the basis of a systematic literature review. According to this 2013 updated guideline, the indications for *HP* eradication therapy have expanded since 1998 [24], and this expansion of target population may have increased the rate of *HP* eradication therapy.

In addition, in the current study, the seronegativity among the subjects who had undergone prior *HP* eradication therapy was 66.2%, which was increased from 59.9% in 2005 and 65.5% in 2011, and this rate has increased significantly over time from 2005 to 2016–2017 (trend *P* < 0.05). Despite the limitation of evaluating the rate of *HP* eradication therapy based on serological tests and self-administered questionnaires, this finding might facilitate a prediction of the changes in the prevalence of GI diseases and increase the understanding of the current public health situation in Korea. The 16-year *HP* eradication rate after standard triple therapy was 74.6% (95% CI, 72.1–77.2%) on an intention-to-treat analysis and 82.0% (95% CI, 80.8–83.2%) on a per-protocol analysis according to a meta-analysis in Korea [25].

Several studies have investigated the risk factors associated with *HP* infection, and the most frequent independent risk factors for *HP* infection are the location of residence in rural areas, poor sanitation, overcrowding, low educational level, and low socioeconomic status [4]. The current study identified older age, medium levels of education, medium levels of income, and male sex as risk factors for *HP* seropositivity. These factors were constant risk factors over time when adjusted for likely confounders.

In terms of biological sex as a risk factor for *HP* seropositivity, most studies reported no significant difference in *HP* seroprevalence between males and females [4]. However, this study showed a strongly increased risk of *HP* seropositivity among men compared with women, which was consistent with the finding in other reports [26–28] including previous reports from South Korea [8–10,28,29]. The importance of this sex-specific difference in the *HP* seroprevalence is still unclear and controversial. However, this finding may partially explain the predominance of male patients with *HP-*related adult diseases in South Korea, including PUD [30] and GC [31].

The strengths of this study were the relatively large sample size (23,770 eligible subjects and 16,885 participants, aged 17–93) and the national scale of the study. Moreover, a comparison of several indexes, including the changes in the rate of *HP* eradication therapy over time, could be performed because of the existence of a series of studies with the same format.

This study also had several limitations. First, it is inevitable that a few false-positive or false-negative results have been included in this study, because the serum level of anti- *HP* IgG antibodies was used as a diagnostic tool. To reduce the false-positive rate, subjects undergoing *HP* eradication therapy were excluded because seroconversion is very slow after *HP* eradication, and the anti-*HP* specific IgG may remain positive even in eradicated cases [32]. However, serologic tests are widely available, noninvasive, inexpensive and appropriate for screening in large epidemiologic studies [12]. Second, the medium-to-high socioeconomic status of our study subjects might also lead to selection bias. Third, we calculated the proportion of subjects receiving *HP* eradication therapy (“the rate of *HP* eradication therapy”) and the prevalence of *HP* seronegativity among the subjects who received *HP* eradication treatment (“putative eradication rate”) using a self-reported questionnaire. Although the questionnaire was administered under the supervision of a well-trained interviewer, there could still be information bias. Fourth, this analysis did not include the possible effects of other environmental factors, such as dietary habits, income spent on healthcare, or national screening strategies, on the change in the *HP* seroprevalence or the change in the rate of *HP* eradication therapy. For the sake of simplicity, this nationwide and multicenter study did not take into dietary habits and other environmental factors.

In conclusion, a downward trend in the seroprevalence of *HP* infection and an increase in the putative *HP* eradication rate were observed over a period of 18 years; both of these trends may influence the spectrum of upper GI disorders and suggest future changes in upper GI disorders in South Korea.

## Supporting information

S1 Fig Comparison of *H*. *pylori* seroprevalences in Jeju by age group in 2011 & 2016–2017. (TIF)

S1 Table STROBE statement—checklist of items that should be included in reports of observational study (cross-sectional study). (DOCX)

## References

[1] McColl KE. Clinical practice. *Helicobacter pylori* infection. *N Engl J Med* 2010;362:1597–1604. 10.1056/NEJMcp1001110 20427808

[2] de Korwin JD, Ianiro G, Gibiino G, Gasbarrini A. *Helicobacter pylori* infection and extra gastric diseases in 2017. *Helicobacter*. 2017;22 (Suppl 1):e12411 10.1111/hel.12411 28891133

[3] Lim JH, Kim N, Lim SH, Kwon JW, Shin CM, Chang YS, et al Inverse relationship between *Helicobacter pylori* infection and asthma among adults younger than 40 years: A cross-sectional study. *Medicine (Baltimore)*. 2016;95:e2609 10.1097/MD.0000000000002609 26937899 PMC4778996

[4] Kim N. Part I Epidemiology 1. Prevalence and Transmission Routes of *H*. *pylori* In: Kim N, editor. Helicobacter pylori. Singapore:Springer; 2016:3–19. 10.1007/978-981-287-706-2_1

[5] Peleteiro B, Bastos A, Ferro A, Lunet N. Prevalence of *Helicobacter pylori* infection worldwide: a systematic review of studies with national coverage. *Dig Dis Sci*. 2014;59:1698–1709. 10.1007/s10620-014-3063-0 24563236

[6] Malaty HM. Epidemiology of *Helicobacter pylori* infection. *Best Pract Res Clin Gastroenterol*. 2007;21:205–214. 10.1016/j.bpg.2006.10.005 17382273

[7] Cave DR. How is *Helicobacter pylori* transmitted? *Gastroenterology*. 1997;113:S9–S14. 10.1016/S0016-5085(97)80004-2 9394753

[8] Kim JH, Kim HY, Kim N, Kim SW, Kim JG, Kim JJ, et al Seroepidemiological study of *Helicobacter pylori* infection in asymptomatic people in South Korea. *J Gastroenterol Hepatol*. 2001;16:969–975. 10.1046/j.1440-1746.2001.02568.x 11595059

[9] Yim JY, Kim N, Choi SH, Kim YS, Cho KR, Kim SS, et al Seroprevalence of *Helicobacter pylori* in South Korea. *Helicobacter*. 2007;12:333–340. 10.1111/j.1523-5378.2007.00504.x 17669107

[10] Lim SH, Kwon JW, Kim N, Kim GH, Kang JM, Park MJ, et al Prevalence and risk factors of *Helicobacter pylori* infection in Korea: nationwide multicenter study over 13 years. *BMC Gastroenterol*. 2013;13:104 10.1186/1471-230X-13-104 23800201 PMC3702482

[11] Von Elm E, Altman DG, Egger M, Pocock SJ, Gøtzsche PC, Vandenbroucke JP for the **STROBE** Initiative. The strengthening the reporting of observational studies in epidemiology (STROBE) statement: guidelines for reporting observational studies. PLoS Med 2007;4: e296 10.1371/journal.pmed.0040296 17941714 PMC2020495

[12] Kim N. Part III Diagnosis 8. Serology In: N, editor. Helicobacter pylori. Singapore:Springer; 2016:113–118. 10.1007/978-981-287-706-2_8

[13] van Der Ende A, van Der Hulst RWM, Roorda P, Tytgat GNJ, Dankert J. Evaluation of three commercial serological tests with different methodologies to assess *Helicobacter pylori* infection. *J Clin Microbiol*. 1999;37:4150–4152. 10565949 10.1128/jcm.37.12.4150-4152.1999 PMC85906

[14] Kim SY, Ahn JS, Ha YJ, Doh HJ, Jang MH, Chung SI, et al Serodiagnosis of *Helicobacter pylori* infection in Korean patients using enzyme-linked immunosorbent assay. *J Immunoassay*. 1998; 19:251–270. 10.1080/01971529808005485 9840297

[15] den Hoed CM, Vila AJ, Holster IL, Perez-Perez GI, Blaser MJ, de Jongste JC, et al *Helicobacter pylori* and the birth cohort effect: evidence for stabilized colonization rates in childhood. *Helicobacter*. 2011;16:405–409. 10.1111/j.1523-5378.2011.00854.x 21923687 PMC3177156

[16] Hooi JKY, Lai WY, Ng WK, Suen MMY, Underwood FE, Tanyingoh D, et al Global prevalence of *Helicobacter pylori* infection: systematic review and meta-analysis. *Gastroenterology*. 2017;153:420–429. 10.1053/j.gastro.2017.04.022 28456631

[17] Pounder RE, Ng D. The prevalence of Helicobacter pylori infection in different countries. *Aliment Pharmacol Ther*. 1995;9(Suppl 2):33–39. 8547526

[18] Kim JI, Kim SG, Kim N, Kim JG, Shin SJ, Kim SW, et al Changing prevalence of upper gastrointestinal disease in 28,893 Koreans from 1995 to 2005. *Eur J Gastroenterol Hepatol*. 2009;21:787–793. 10.1097/MEG.0b013e32830e285a 19404205

[19] Bae S, Kim N, Kang JM, Kim DS, Kim KM, Cho YK, et al Incidence and 30-day mortality of peptic ulcer bleeding in Korea. *Eur J Gastroenterol Hepatol*. 2012;24:675–682. 10.1097/MEG.0b013e3283525a56 22441511

[20] Bae S, Shim KN, Kim N, Kang JM, Kim DS, Kim KM, et al The incidence and short-term mortality of perforated peptic ulcer in Korea: a population-based study. *J Epidemiol*. 2015;22:508–516. 10.2188/jea.JE20120056 22955110 PMC3798562

[21] Korean H. *pylori* Study Group. Diagnosis and treatment of *Helicobacter pylori* infection in Korea. *Korean J*. *Gastroenterol*.1998;32:275–289.

[22] Kim N, Kim JJ, Choe YH, Kim HS, Kim JI, Chung IS, et al [Diagnosis and treatment guidelines for *Helicobacter pylori* infection in Korea]. *Korean J*. *Gastroenterol*. 2009;54:269–278. 10.4166/kjg.2009.54.5.269 19934608

[23] Kim SG, Jung HK, Lee HL, Jang JY, Lee H, Kim CG, et al Guidelines for the diagnosis and treatment of Helicobacter pylori infection in Korea, 2013 revised edition. J Gastroenterol Hepatol. 2014;29:1371–1386. 10.1111/jgh.12607 24758240

[24] Lee JY. Part VII Treatment 48. Treatment guidelines In: Kim N. editor. Helicobacter pylori. Singapore: Springer;2016:487–493. 10.1007/978-981-287-706-2_48

[25] Lee JY. Part VII Treatment 41. Triple therapy In: Kim N. editor. Helicobacter pylori. Singapore: Springer;2016:427–436. 10.1007/978-981-287-706-2_41

[26] Replogle ML, Glaser SL, Hiatt RA, Parsonnet J. Biologic sex as a risk factor for *Helicobacter pylori* infection in healthy young adults. *Am J Epidemiol*. 1995;142:856–863. 10.1093/oxfordjournals.aje.a117725 7572962

[27] Everhart JE, Kruszon-Moran D, Perez-Perez GI, Tralka TS, Mcquillan G. Seroprevalence and ethnic differences in *Helicobacter pylori* infection among adults in the United States. *J Infect Dis* 2000;181:1359–1363. 10.1086/315384 10762567

[28] de Martel C, Parsonnet J: *Helicobacter pylori* infection and gender: a meta-analysis of population-based prevalence surveys. *Dig Dis Sci*. 2006;51:2292–2301. 10.1007/s10620-006-9210-5 17089189

[29] Sung KC, Rhee EJ, Ryu SH, Beck SH. Prevalence of Helicobacter pylori infection and its association with cardiovascular risk factors in Korean adults. *Int J Cardiol*. 2005;102:411–417. 10.1016/j.ijcard.2004.05.040 16004885

[30] Kim JJ, Kim N, Park HK, Jo HJ, Shin CM, Lee SH, et al [Clinical characteristics of patients diagnosed as peptic ulcer disease in the third referral center in 2007]. *Korean J Gastroenterol*. 2012;59:338–346. 10.4166/kjg.2012.59.5.338 22617527

[31] Kim JY, Lee HS, Kim N, Shin CM, Lee SH, Park YS, et al Prevalence and clinicopathologic characteristics of gastric cardia cancer in South Korea. *Helicobacter*. 2012;17:358–368. 10.1111/j.1523-5378.2012.00958.x 22967119

[32] Lee JH, Kim N, Chung JI, Kang KP, Lee SH, Park YS, et al Long-term follow up of *Helicobacter pylori* IgG serology after eradication and reinfection rate of *H*. *pylori* in South Korea. *Helicobacter*. 2008;13:288–294. 10.1111/j.1523-5378.2008.00616.x 18665939

