# Supplementary figures and images for: Trends in the seroprevalence of Helicobacter pylori infection and its putative eradication rate over 18 years in Korea: A cross-sectional nationwide multicenter study
Source: PLoS One. 2018 Oct 17;13(10):e0204762. doi: 10.1371/journal.pone.0204762 (PMC6192591; doi:10.1371/journal.pone.0204762)

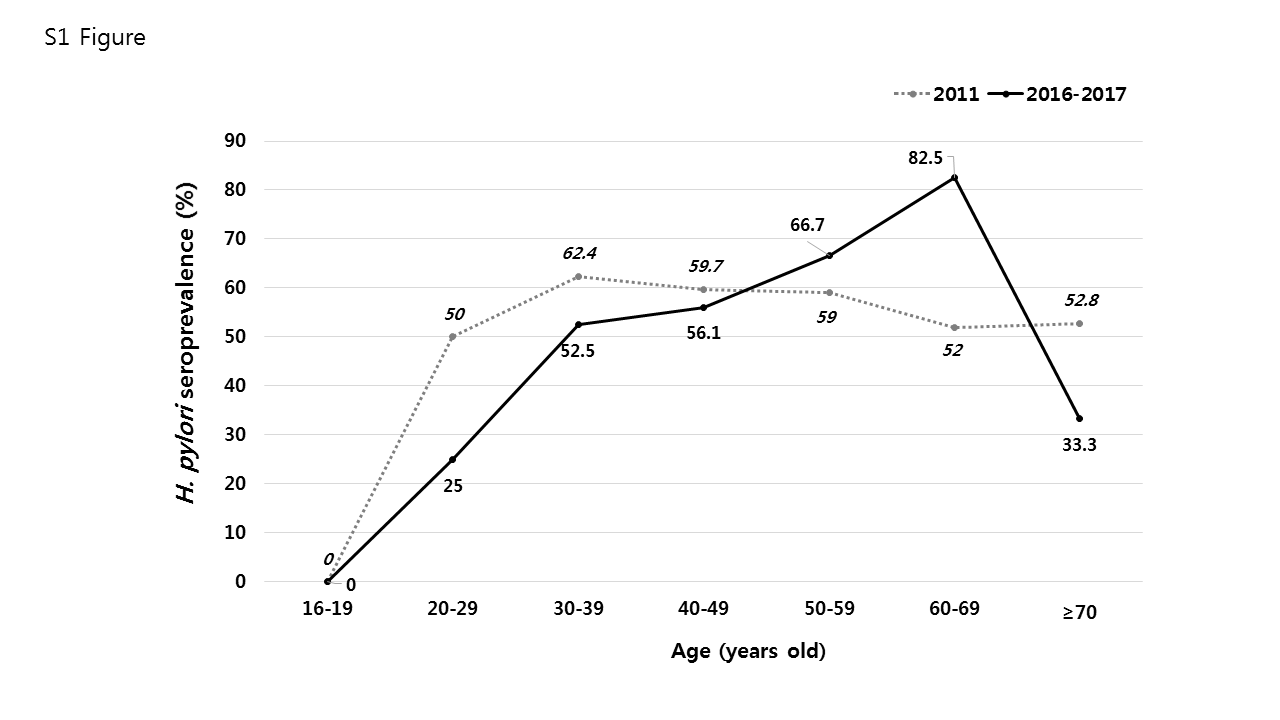

Supplement: S1 Fig — (TIF) [file pone.0204762.s001.tif]
